# Surface modification of plasmonic noble metal–metal oxide core–shell nanoparticles

**DOI:** 10.1039/c9na00581a

**Published:** 2019-10-30

**Authors:** Somayeh Talebzadeh, Clémence Queffélec, D. Andrew Knight

**Affiliations:** Department of Biomedical & Chemical Engineering & Sciences, Florida Institute of Technology 150 West University Boulevard Melbourne Florida 32901 USA aknight@fit.edu; Nantes Université, CNRS, CEISAM, UMR 6230 F-44000 Nantes France clemence.queffelec@univ-nantes.fr

## Abstract

A comprehensive survey on the methods for the surface modification of plasmonic noble metal–metal oxide core–shell nanoparticles is presented. The review highlights various strategies for covalent attachment and electrostatic binding of molecules and molecular ions to core–shell nanoparticles with a focus on plasmonically active silver and gold nanoparticles encapsulated by SiO_2_ and TiO_2_ shells.

## Abstract 2

A comprehensive survey on methods for surface modification of noble metal–metal oxide core–shell nanoparticles is presented and highlights various strategies for binding of molecules and molecular ions to core–shell nanoparticles.

## Graphical abstract

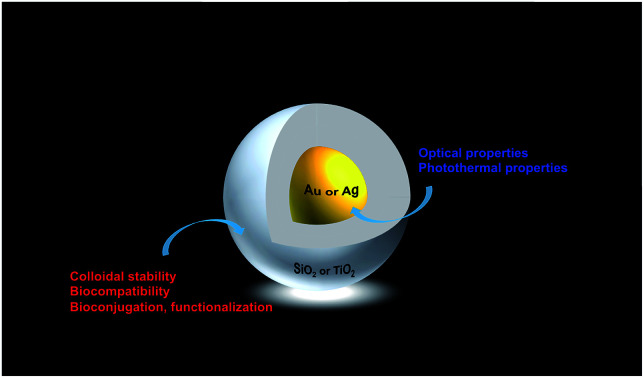

## 1. Introduction

Recently, the development of nanomaterials and specifically nanoparticles (NPs) has been the center of focus due to their unique properties when compared to the corresponding bulk materials. The unique property of metallic NPs known as localized surface plasmon resonance (LSPR) has been exploited for enhancing the optical properties, photothermal heating, stability and biocompatibility of nanoparticles. Noble metal@metal oxide core–shell nanoparticles including M@SiO_2_ prepared with a noble metal core have received significant attention with a broad range of applications in many different fields, such as biomaterials, sensing, dye-sensitized solar cells, catalysis and photocatalysis.^1–5^ In addition, the oxide shell around the metal nanoparticles provides robustness and chemical stability and has the capability of improving the reactivity of the surface and thus providing accessible sites for further conjugation of molecular structures and larger platforms. Noble metal@TiO_2_ core–shell nanoparticles have also received attention with a focus on photochemical surface plasmon resonance assisted catalysis. Covalent grafting of molecules to TiO_2_ is ideally achieved using organophosphonic acids RPO_3_, which results in a hydrolytically stable surface coating.^6^

Novel optical properties, biocompatibility and use in biomedicine are the most interesting features and applications of the core–shell nanoparticles.^2,7,8^ Although some applications have already been established there is still a need for providing new biosensors, biomarkers or diagnostic imaging which are of paramount importance for rapid detection and selectivity of various biomolecules such as antibodies, drugs, DNA, and lipids. These applications are made possible due to the tunable shape, size of metallic NPs and the possibility of surface grafting.^9,10^ Shell-isolated nanoparticles have been applied in surface-enhanced Raman scattering (SERS) and fluorescence studies and has also received a great deal of attention due to its sensitivity in chemical and biomedical analysis. However, the sensitivity and reproducibility depends on the active substrate. Thus the grafting or bonding of SERS reporters is of paramount importance.^2,11,12^

Major developments have been made in metal-enhanced fluorescence (MEF) in which fluorophores interact with metallic NPs increasing photostability. Studies have shown that fluorophores embedded within the core–shell around metallic NPs show a significant fluorescence enhancement due to close proximity of the fluorophores to the metallic core but avoid quenching of the fluorescence. The fluorescence enhancement depends on the particle size, shape, and the distance between the fluorophore and the metal surface.^13–16^ MEF have numerous applications in nanomedicine, analytical chemistry, and biology but this technique requires the building of nanostructures with control of size, shape of the core–shell NPs, and the surface attachment of molecules.

Both the SERS and MEF methods have advantages such as detecting trace amounts of analyte for SERS as for enhanced-fluorescence the advantage relies in the high sensitivity and quick display of variations in concentration of analytes. The combination of both techniques could be a promising way to extend applications in biodetection and bioimaging, but those detection methods require surface grafting chemistries.

This review describes contemporary synthetic approaches for the introduction of a chemical modifier on the surface of noble metal/metal oxide core–shell NPs introduced either by covalent binding or electrostatic interaction and a summary is shown in Table 1. It is worth noting that the synthesis and characterization of a wide variety of *non-noble* metal@metal oxide nanoparticles have been prepared, characterized and used in miscellaneous applications (*e.g.* Ni@NiO and Fe@Fe_2_O_3_). However the vast majority of these materials do not possess plasmonic properties or significant molecular surface modification, and this field has been adequately reviewed.^8,17,18^

**Table 1:** Summary of surface modification methods

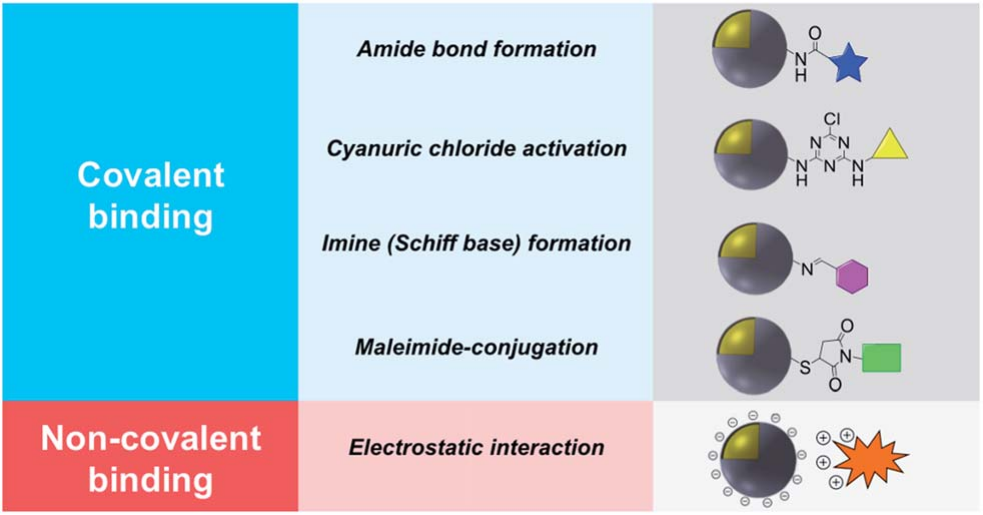

## 2. Surface modification using covalent binding

**Fig. 1:**
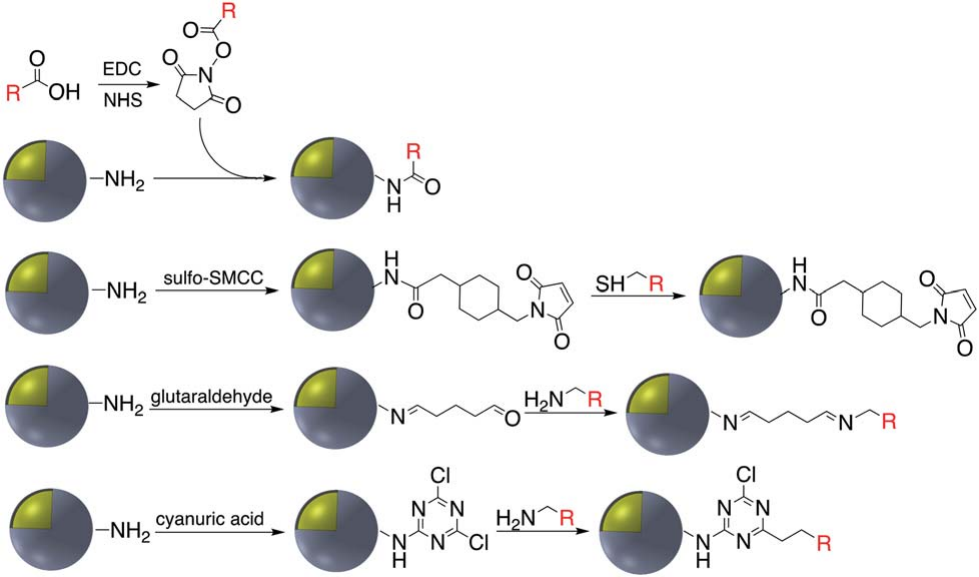
Examples of surface modification of core–shell M@SiO_2_ (M = Au, Ag) using organosilane reagents.

Covalent binding is mostly discussed in regard to silica-coated noble metals and the wide variety of silane reagents available for coupling chemistry. Silane linkers can be condensed onto the surface of a silica shell through the alkoxysilane groups, providing sites for further immobilization of biomolecules, therapeutics, and coordination complexes.^19–21^ Since biomolecules and organic ligands contain functional groups such as amine, carboxylic acid and thiol, common conjugation reactions have been used for covalent attachment (Fig. 1).

### 2.1 Amide bond formation

**Fig. 2:**
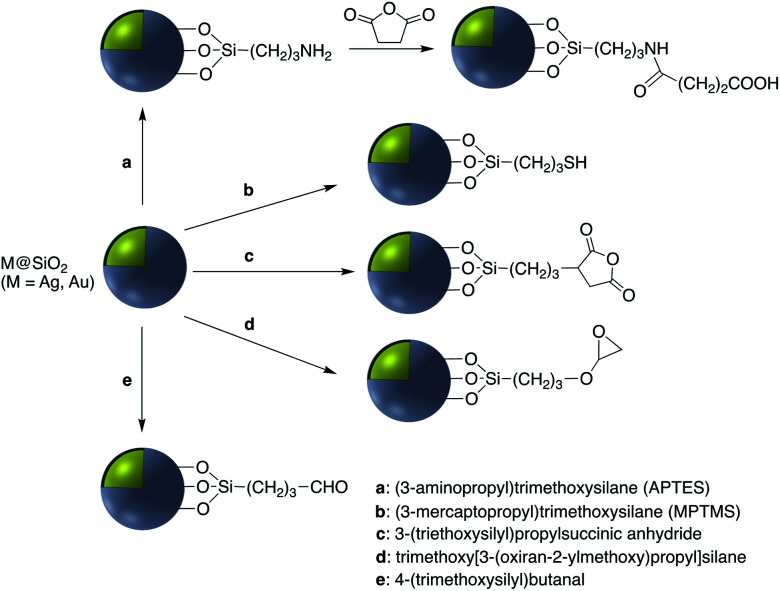
Coupling strategies for amine-functionalized core–shell nanoparticles.

Carbodiimide crosslinking chemistry, using water soluble 1-ethyl-3-(3-dimethylaminopropyl)carbodiimide (EDC) is the most convenient and efficient method to bind chemical modifiers onto the surface of silica coated noble metal nanoparticles using amide bond formation (Fig. 2).

The first reaction of the carboxylate group whether it is on the silica shell surface or part of the chemical modifier is with EDC, followed by a reaction with *N*-hydroxysuccinimide (NHS) forming a stable intermediate which then reacts with a suitable amine.^22,23^ For example, Li *et al.* synthesized Au@SiO_2_ with various morphologies of the core for further functionalization with H_2_N–DNA *via* a standard EDC/NHS coupling. The resulting conjugate was applied as a surface-enhanced Raman scattering (SERS) probe for monitoring DNA hybridization.^24^ Using the same strategy, ssDNA was immobilized onto the surface of Ag@SiO_2_@SiO_2_ core–shell nanoparticles in which the outer silica layer was doped with energy donor ligand complex RuBpy. ssDNA was then hybridized with a prostate specific antigen (PSA)-aptamer and applied as a fluorescence sensor for quantitative detection of PSA.^25^

**Fig. 3:**
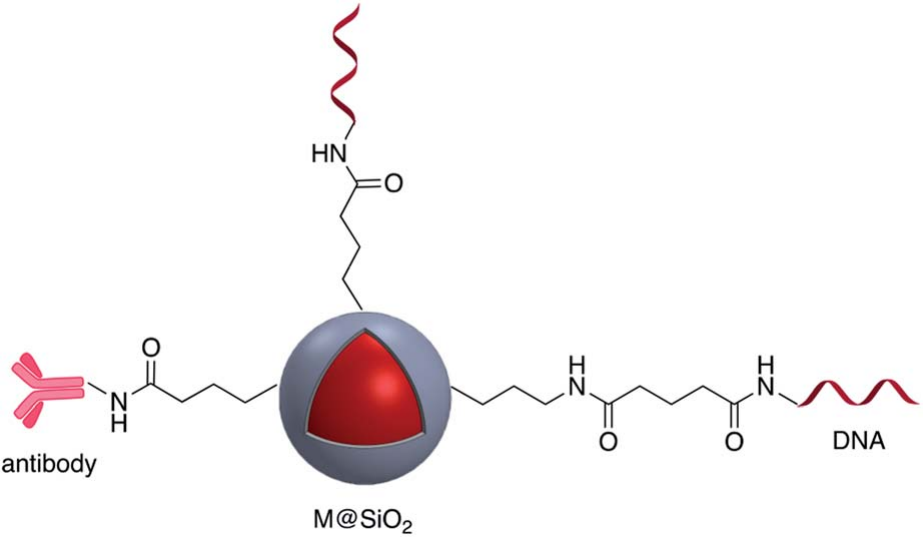
Antibody and DNA modified core–shell nanoparticles (M = Au, Ag).

Brouard *et al.* used the same method to prepare Ag@SiO_2_–DNA for human SRY gene detection assisted with plasmonic enhanced fluorescence of the silver core. The outer silica shell was covalently incorporated with eosin dye prior to bioconjugation.^26,27^ The same research group developed a very sensitive metal-enhanced fluorescence (MEF) sensor for DNA detection using immobilization of DNA and a cationic polymeric transducer, onto the surface of Ag@SiO_2_.^26^ In another study, a DNA-functionalized gold nanostar@Raman-reporter@SiO_2_ sandwich structure was developed and applied in the SERS detection of trace amounts of heavy metals in human saliva, specifically silver and mercury leached from dental fillings.^28^ Guo *et al.* Ag@SiO_2_ nanoparticles with different shell thicknesses were synthesized *via* modified Stöber method and rhodamine B isothiocyanate was covalently bound onto the surface.^29^ Examples of antibody and DNA modified core–shell NPs are shown in Fig. 3.

**Fig. 4:**
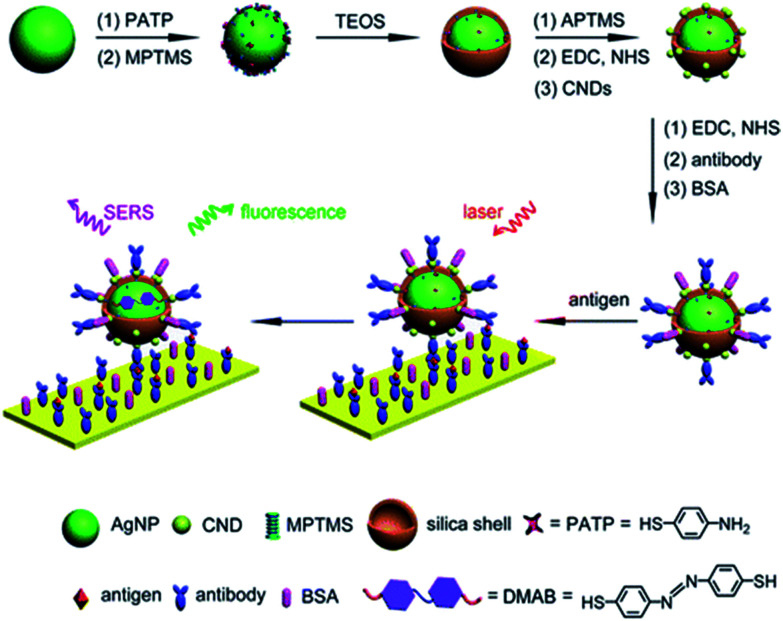
Carbon nanodot-decorated Ag@SiO_2_ NPs for fluorescence and SERS immunoassays. Reprinted (adapted) with permission from ref. 30. Copyright 2016 American Chemical Society.

Fluorescent carbon nanodots were covalently attached by EDC/NHS coupling reaction onto Ag@SiO_2_ NPs and then subsequently employed for conjugation of antibodies on the surface of the hybrid nanomaterial (Fig. 4).^30^ Similarly EDC coupling was used to conjugate monoclonal anti-Zika virus NS_1_ antibodies to gold shell-isolated nanoparticles (Au-SHINs) for Zika virus detection using SERS.^31^

Carboxylate groups on the surface of Au-nanostar@SiO_2_ were activated through an EDC/NHS process for subsequent reaction with an amine terminated CEA monoclonal antibody (CEA mAb) and mouse anti-cancer CA27-29 (CA27-29 mAb). The modified nanoparticle was used as a SERS nanoprobe for the detection of cancer markers using plasmon-enhanced Raman spectro-immunoassay.^32^ The same approach was applied to conjugate immunoglobulin-G (lgG) onto the surface of fluorescent Ag@SiO_2_ and studied using electron microscopy.^33^ Quyen *et al.* prepared a SERS probe using Ag@SiO_2_, loaded with rhodamine 6G (R6G) and carcinoembryonic antibody. Ag@SiO_2_–NH_2_ was first modified with poly(ethyleneglycol) bis(carboxymethyl)ether (PEGC) to provide carboxylate groups for EDC coupling.^34^ In a recent study by Xu *et al.*, Ag@SiO_2_@SiO_2_–RuBpy nanoparticles with 3-fold photoluminescence enhancement were prepared and modified with anti-prostate specific antigen (PSA) antibody using EDC/NHS reaction and activation of –COOH moieties on the surface of nanocomposites. The platform showed promising results based on metal-enhanced fluorescence for PSA detection and early diagnosis of prostate cancer.^35^

**Fig. 5:**
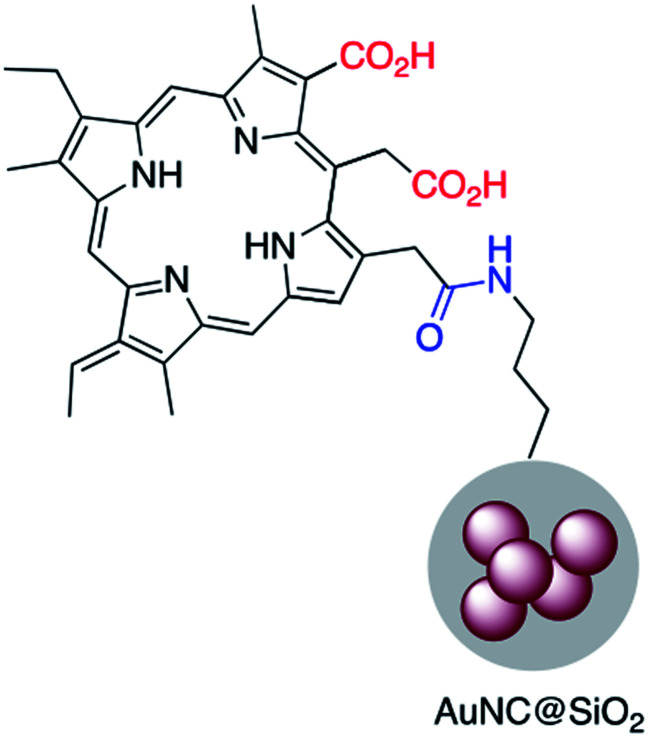
A gold–silica core–shell nanocluster modified with a porphyrin based photosensitizer.

Chlorine e6 (Ce6) is a well-known photosensitizer and was conjugated to silica-coated gold nanoclusters (NCs) to provide a novel photo-theranostic agent in which both the fluorescence of Ce6 and plasmon luminescence of the gold NCs was exploited for sub-cellular characterization. Conjugation was conducted using a standard EDC/NHS reaction, and activated carboxylic acid groups of Ce6 were covalently linked to NH_2_–Ag@SiO_2_ (Fig. 5).^36^ A similar study was carried out for surface modification of a silica coated triangular nanoprism with Ce6 to prepare a plasmon-enhanced fluorescent probe for pyrophosphate (PPi) detection with a detection limit of 0.2 μM. In this study, HOBT was used instead of NHS to synthesize the stable intermediate.^37^ Similarly, Wang *et al.* immobilized *meso*-tetra(4-carboxyphenyl)porphyrin (TCPP) on the surface of AuNR@SiO_2_ for fluorescence detection of PPi in aqueous solutions.^38^ Similarly, TCPP–copper complexes were attached to AuNR@SiO_2_ and used for the fluorescence detection of hydrogen sulfide.^39^ A hybrid system consisting of both Ag@SiO_2_ and gold nanoclusters was used for the simultaneous detection of Cu^2+^ and PPi^40^ and Au@Ag@SiO_2_ core–shell nanostructures were used as an ultrasensitive SERS immunoassay of alpha-fetoprotein.^41^

**Fig. 6:**
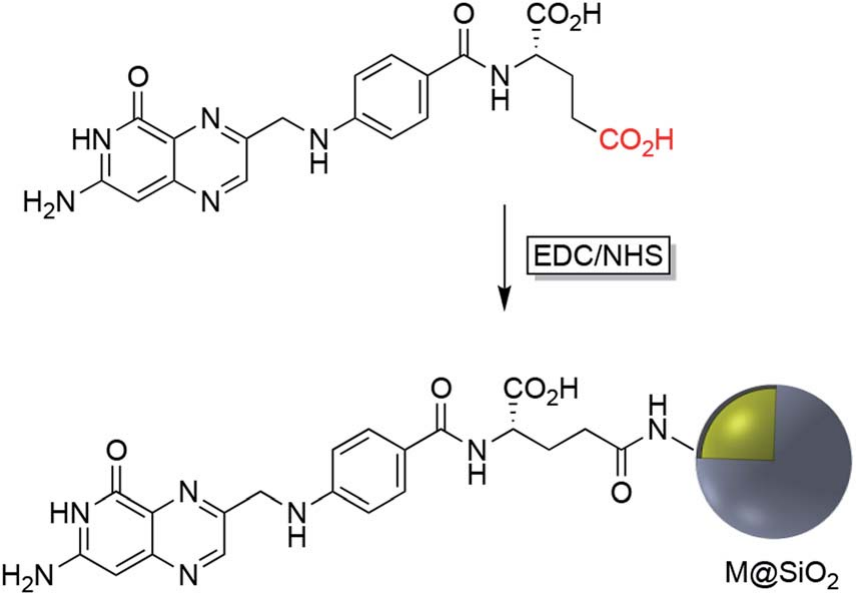
Synthesis route to folic acid-conjugated silica-coated plasmonic nanoparticles (M = Ag, Au).

EDC/NHS crosslinking chemistry has been used also for the immobilization of folate on the surface of Ag or Au nanoparticles coated with silica. The folic acid/folate receptor interaction can be targeted for imaging cancer cells by the attachment of imaging-probe molecules to the folate moiety (Fig. 6). Folic acid has a carboxylate group in its structure that can couple with amine-modified core–shell nanoparticles using EDC. Folic acid may be functionalized with molecular probes prior to surface attachment. Folate modified nanocomposites have been applied in a variety of fields including drug delivery, tumor radiotherapy, cancer treatment, photothermal therapy, and bioimaging.^42–50^

**Fig. 7:**
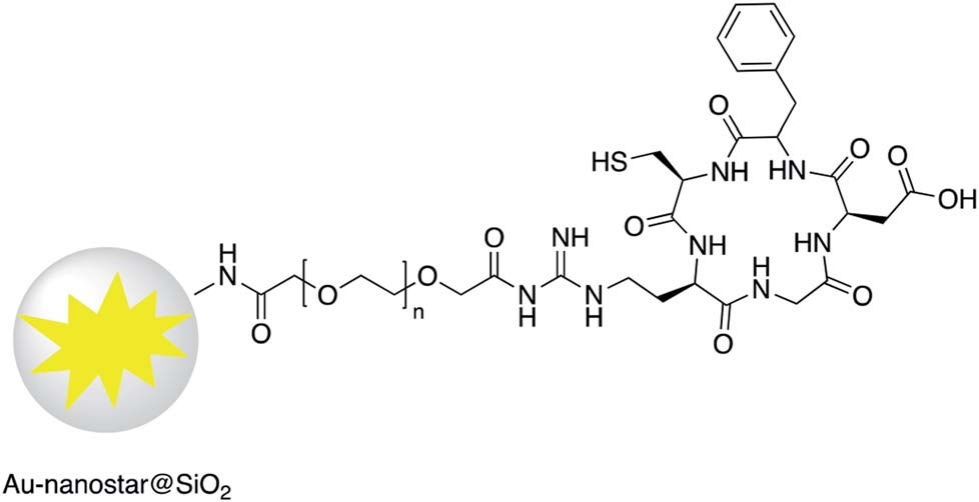
Au-nanostar@SiO_2_ modified with RGD peptide.

Increased temperature resulting from the surface plasmon resonance (SPR) of AuNR@SiO_2_–tLyP-1, loaded with camptothecin (CPT), was applied for photothermal therapy of cancer cells. tLyP-1 peptide, which contains a primary amine, was covalently linked to the surface of a silica shell using EDC and NHS reagents.^51^ Core–shell Au-nanostar@SiO_2_ was modified with c(RGDfk) peptide for SERS mapping and diagnosis of breast cancer cells (Fig. 7). Amine groups on the surface of a core–shell nanoparticle were first linked to poly(ethylene glycol)bis(carboxymethyl)ether (5000 MW) *via* EDC/NHS coupling. Conjugation of c(RGDfk) to the free carboxyl group of PEG was also achieved *via* EDC/NHS coupling.^52^

**Fig. 8:**
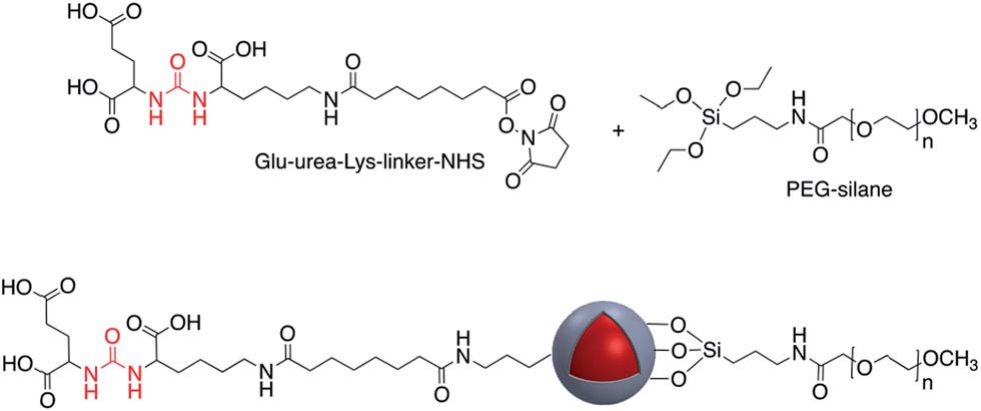
Core–shell nanoparticles modified with glu-urea-Lys-linker-NHS.

Liu *et al.* designed a photothermally responsive nanoprobe based on Edman degradation which can be used for tumor-targeted bioimaging and drug delivery. In this strategy, Au-nanorod@SiO_2_ was first modified with thiocyanate. Glu(Cy5.5)-c(RGDyk) which contains primary amine groups then reacts with the thiocyanate functionality. The final product showed a high capacity for the release of Cy5.5–NH_2_ even with low energy power irradiation, and under very mild conditions, *via* AuNP core surface energy transfer (NSET).^53^ There are several other examples where EDC/NHS coupling has been used to modify the surface of Au@SiO_2_ and Ag@SiO_2_ with different molecules such as glucose oxidase enzyme (GOx), 4-azidobenzoic acid, and oligothiophene.^53–56^ Lee *et al.* used EDC/NHS coupling to modify the surface of Ag@SiO_2_ core–shell nanoparticles with oligothiophene. Polymer stabilized nanoparticles were then incorporated into the structure of photovoltaic cells to enhance their ability for solar energy harvesting.^56^ Sotiriou and coworkers investigated the grafting of gallic acid onto the surface of Ag@SiO_2_. Gallic acid is a natural antioxidant that can perform proton-coupled electron transfer and the modified Ag@SiO_2_ exhibited an enhanced plasmonic resonance at near-IR wavelengths which is due to the hot-spot formation mediated by the gallic acid. The antioxidant modified nanoparticles also possessed radical scavenging properties.^57,58^ Amide bond formation was also used for grafting the surfaces of Ag@SiO_2_ and Au@SiO_2_ with different polyethylene glycol (PEG) molecules to increase their stability and dispersibility.^59–61^ Cy7 NHS ester was chemically bound to the surface of gold nanopyramids coated with silica as an infrared-fluorescent biosensor for detection of PPi.^62^ Li *et al.* functionalized the surface of Au-nanostar@SiO_2_–NH_2_ with a urea-based molecule as a SERS agent for prostate cancer targeted imaging (Fig. 8).^63^ In a recent investigation, the synthesis of a new selective plasmonic sensor for colorimetric detection of glucose was reported with a detection limit of 2.06 × 10^−5^. This method is significantly more sensitive compared to the commercially available glucose kits. In this method freshly prepared NH_2_ functionalized Ag@SiO_2_ core–shell nanoparticles were reacted with activated –COOH moieties of acrylic acid using EDC/NHS reagents. Aggregation and decrease in the surface plasmon absorption of the modified nanocomposites after polymerization of acrylic acid was used to detect the glucose. Polymerization of the acrylic acid was induced using GOx in the presence of FeCl_2_.^64^

EDC/NHS coupling has been used as an effective method for the attachment CdTe quantum dots (QD) to the surface of plasmonic nanoparticles coated with a silica shell. Zhu *et al.* use silica-coated gold nanoparticles to enhance the fluorescence properties of CdTe QD and improve the sensing ability for mercury(ii) using Au@SiO_2_. In this report Au@SiO_2_ NPs were functionalized with –NH_2_ using APTES and then CdTe QD–COOH was activated through the EDC/NHC coupling to give Ag@SiO_2_@CdTe QD^65^ and Ag@SiO_2_@QD.^66^

In another study by Gontero and co-workers, Au@SiO_2_ with different shell thicknesses of silica, and functionalized with Rhodamine B, was used as a nanosensor for bacterial tracking and imaging based on metal-enhanced fluorescence. In this work, Rhodamine B was initially conjugated to (3-aminopropyl)trimethoxysilane (APS) using EDC/NHS coupling and then Rhodamine–APS was reacted with Ag@SiO_2_.^67^

EDC/NHS coupling was also used to bind hemoglobin onto gold NPs entrapped in an aminated titanium dioxide shell.^68^ This platform was used as a biosensor for the detection of hydrogen peroxide that is commonly used as a food additive and preservative. Gontero *et al.* grafted rhodamine molecules onto gold core–shell NPs with APS *via* EDC/NHS to afford luminescent platforms for bacterial detection.^67,69^ The luminescent platform was deposited onto *E. coli* and clear bacterial images were obtained by laser fluorescence microscopy however in the absence of nanoparticles no image was observed. This nano-imaging platform based on ultraluminescent core–shell nanoparticles was then applied using a microfluidic system, a less-time consuming bioanalytical method. Amide-bond formation has also been applied to silver–titania core–shell functionalization. For example, Wan *et al.* investigated the antibacterial activity of vancomycin-functionalized Ag@TiO_2_ under UV light irradiation.^70^ The surface of the Ag@TiO_2_ was first coated with dopamine to provide primary amine groups for further reaction with the homobifunctional cross-linker suberic acid bis(*N*-hydroxysuccinimide ester) (DSS). Then, activated Ag@TiO_2_ was reacted with the antibiotic vancomycin *via* the free amine groups. The resulting antibiotic functionalized core–shell nanoparticles were shown to selectively photokill pathogenic bacteria.

### 2.2 Cyanuric chloride activation

**Fig. 9:**
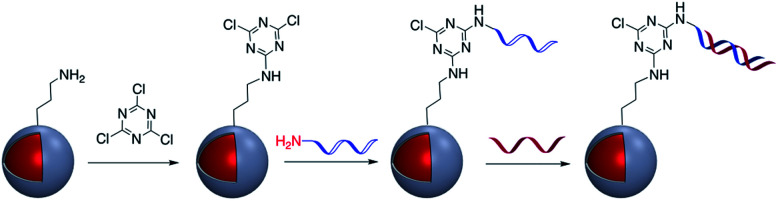
Preparation of DNA-modified Au@SiO_2_ core–shell nanoparticles using cyanuric acid coupling.

The use of a cyanuric acid linker to modify the surface of M@SiO_2_ coated with amine-functionalized molecules has been investigated in recent years (Fig. 9).^71,72^

For example, Zhou *et al.* developed a SERS nanoprobe for the imaging of latent fingerprints (LFPs). Amino-modified Au@SiO_2_ was reacted with 2,4,6-trichloro-1,3,5-triazine (cyanuric acid) to provide a triazine-activated surface and then modification was accomplished with the further reaction of an amine-terminated aptamer with the activated silica shell *via* nucleophilic substitution.^73^ In addition to this example, the method was used to modify Ag@SiO_2_ with DNA aptamers to exploit the effect of the plasmonic core in the detection of several biomolecules and inorganic species such as recombinant hemagglutinin (rHA) proteins, adenosine triphosphate (ATP), and trace amounts of mercury.^74–77^

### 2.3 Imine (Schiff base) formation

**Fig. 10:**
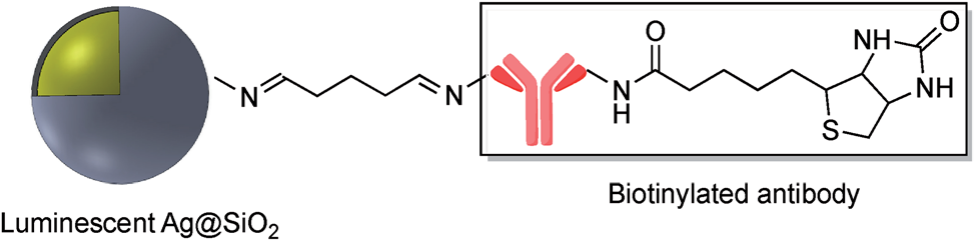
Biotinylated antibody grafted onto the surface of core–shell Ag@SiO_2_.

Schiff bases (general formula R′–N=CR_2_) are readily assembled from an organic amine and a suitable aldehyde. Schiff base formation has been used to link different molecules with primary amine moieties onto the surface of M@SiO_2_. This method has been carried out by using aldehyde modified core–shell nanoparticles. M@SiO_2_ nanoparticles functionalized with aldehydes have been attached to NH_2_–DNA, and the resulting conjugates have been used in biomedical applications.^78–81^ A variety of SERS biosensors were fabricated by the immobilization of anti-h-IgG on the surface of amine-functionalized Ag@SiO_2_ and Au@SiO_2_. The surface of nanocomposites was coated with glutaraldehyde (GA) prior to the incorporation of the amine functionalized antibody.^82–86^ For example, Wang *et al.* used imine formation to prepare a colorimetric biosensor for the detection of h-IgG antigen and Gong and co-workers used a similar strategy for covalent attachment of anti-h-IgG onto the surface of Ag@SiO_2_. A fluorescent dye-doped, silica-coated gold nanoparticle aggregate, modified with anti-h-IgG antibody was employed as a dual mode biosensor and the particularly intense surface plasmon band and SERS activity of the modified nanocomposite, in combination with the conjugated antibody was used in multiplex biodetection.^85^ Wei *et al.* also reported the improvement of a biological immunoassay using Ag@SiO_2_ modified with anti-h-IgG antibody incorporating a Raman reporter.^84^ Biotinylated antibody was grafted onto the surface of Ag@SiO_2_ using the same method, and modified nanocomposites, which were previously doped with the lanthanide metal europium in the form of a chelate complex (BHHCT-Eu-DPBT), were found to be suitable for fluorescence bioimaging of cells (Fig. 10).^87,88^

**Fig. 11:**
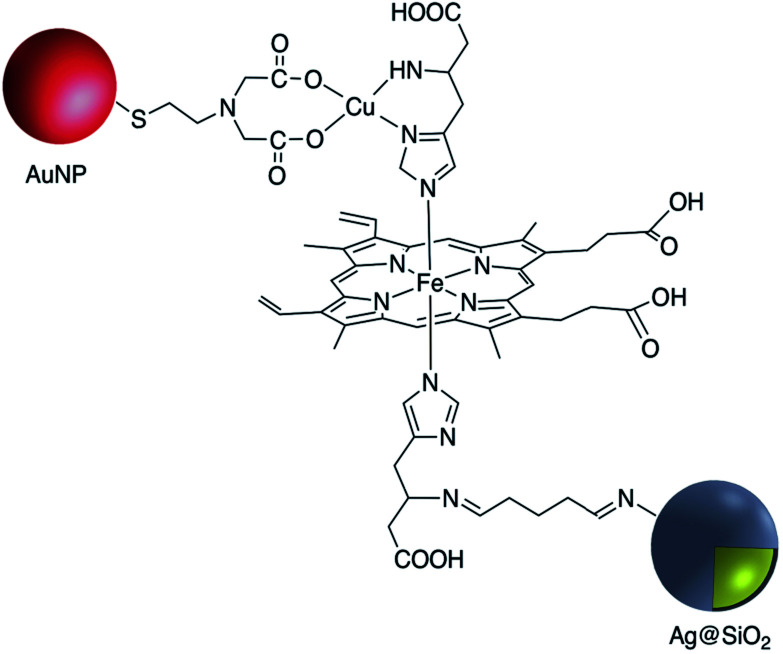
Myoglobin-modified Ag@SiO_2_.

Yin *et al.* also used this technique for the modification of Ag@SiO_2_ with the amine-reactive 4-formylphenylboronic acid. Amino groups on the surface of Ag@SiO_2_ were reacted with the CHO functional group to give a stable C=N imine bond. The affinity of the boronic acid –B(OH)_2_ functional group towards the sugar sialic acid was taken advantage of in the preparation of a robust ion imprinted platform for SERS imaging of cancer cells and tissue^89^ and Narayan *et al.* immobilized the protein bovine serum albumin (BSA) onto the surface of Au@SiO_2_@NH_2_ using a glutaraldehyde cross-linker, again exploiting the stability of the imine linkage and reactivity of amine and aldehyde moieties. BSA was also conjugated through EDC/NHS coupling, and electrostatic absorption and the modified Au@SiO_2_ nanoparticles were compared based on the differences in their physical and chemical properties.^90^ The metalloprotein myoglobin (Mb) which contains at least four histidine (His) residues was covalently bound to amine-Ag@SiO_2_ SERS tags through glutaraldehyde (GA) conjugation. Mb-conjugated Ag@SiO_2_ was further mixed with a colloidal solution of functionalized AuNP with 2-(iminodiacetic acid)ethanethiol (IDA) and applied for protein detection in biological studies (Fig. 11).^91^ Xia *et al.* prepared Ag@SiO_2_ functionalized with anti-HER2 using the Schiff base formation reaction. In this method H_2_N-antibody was covalently conjugated to oxidized dextran 500 modified Ag@SiO_2_. The resulting conjugate, which contains 4-MBA as a Raman reporter was utilized as a SERS tag for imaging cancer cells.^92^ In summary, imine (Schiff base) formation has provided a suitable technique for the attachment of molecules to the surface of core–shell metal–metal oxide nanoparticles and is only limited by the synthetic availability of the appropriate amine and aldehyde precursors.

### 2.4 NHS ester–maleimide-conjugation

Maleimide derivatives can be used as cross-linkers to introduce thiol and amine terminated biomolecules such as oligonucleotides and proteins onto the surface of silica coated noble metal core–shell nanoparticles.^93–97^

Sui *et al.* used sulfosuccinimidyl-4-(*N*-maleimidomethyl)-cyclohexane-1-carboxylate (sulfo-SMCC) to prepare a stable maleimide-activated Ag@SiO_2_ that was subsequently conjugated to thiol functionalized SH-DNA exploiting the facile reactivity of the maleimide group with thiols. That platform was then used for heavy metal and protein detection.^98,99^ Using a similar strategy, Cerruti and co-workers coated the surface of Au@SiO_2_ with SH-ssDNA for use as a SERS probe for DNA detection.^97^ Amine functionalized Au-nanocage@SiO_2_ was modified with thiol ended tag peptides using sulfo-SMCC cross linker which were then used as versatile SERS nanoprobes for imaging, drug delivery and photothermal therapy^95^ and DNA was successfully attached to the surface of silver nanoprisms coated with silica by using a succinimidyl 4-(*p*-maleimidophenyl)butyrate (SMPB) cross-linker (Fig. 13).^100^

**Fig. 12:**
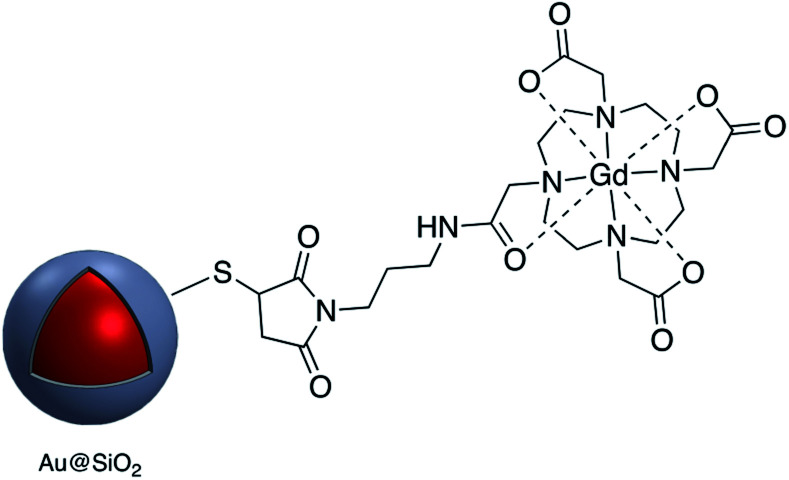
Gd-DOTA-modified core–shell nanoparticle.

Kustner and co-workers modified the surface of silica capsulated gold and silver nanoparticles with monoclonal antibodies for antigen recognition. In this method amine-functionalized core–shell nanoparticles were conjugated to a maleimide bifunctional cross-linker with a polyethylene glycol spacer and then an antibody modified with thiol groups was bonded to the functionalized core–shell nanoparticle.^94^ The paramagnetic contrast agent consisting of a macrocyclic chelate ligand–lanthanide complex 1,4,7,10-tetraazacyclododecane-1,4,7,10-tetraacetic acid (DOTA)-Gd^3+^ was conjugated onto the surface of Au@SiO_2_, also *via* a maleimide linkage (Fig. 12). The modified core–shell nanoparticles, containing Raman receptors were found to be useful in MRI imaging.^96^

**Fig. 13:**
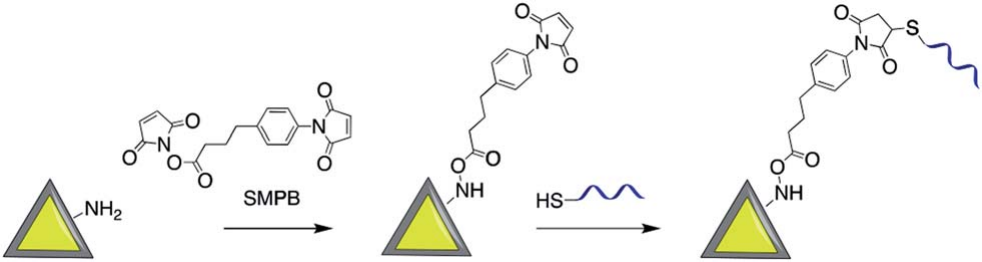
Synthesis of Ag-nanoprism@SiO_2_ modified with DNA assembled using maleimide conjugation.

Biotin was conjugated to the surface of amine and thiol functionalized-Ag@SiO_2_ using the reagents maleimide (MAL)-discretePEG_3_-biotin and NHS-d-PEG_3_™—biotin. Fluorescence enhanced properties of the modified tags showed promise for biomedical imaging.^101^

**Fig. 14:**
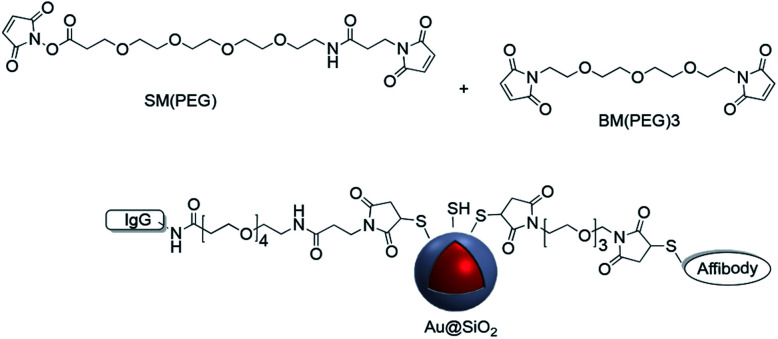
Maleimide coupling used to modify the surface of Au@SiO_2_ with two separate biomolecules.

CD4 is a 51 kDa surface marker expressed on T-cells, and antibodies to CD4 can be used in the study of signal transduction in cells. Dong *et al.* grafted CD4 antibodies onto the surface of Ag@SiO_2_ nanoparticles using sulfo-SMCC which were then used for cell labeling.^102^ Maleimide–PEG–NHS molecules with different molecular weights were used to prepare active Au-nanorod@SiO_2_ surfaces for further conjugation to peptides with thiol groups such as tLyP-1 peptide and SH-RGD.^103,104^ The modified nanostructures were used for targeting imaging, photothermal therapy (PPT) and drug delivery. Although the previous examples all involve surface modification with a single linker, it is possible to use more than one linker for conjugation. For example, Jokerst *et al.* carried out the dual surface coating of Au@SiO_2_ with 1,11-bis-maleimido-triethyleneglycol (BM(PEG)) and succinimidyl-ester (SM(PEG)). The modified shell was conjugated to a variety of biomolecules with sulfhydryl and amine moieties highlighting the versatility of the method (Fig. 14).^105^

A similar technique was employed for coupling amine-end capped antibodies to Au@SiO_2_ nanoparticles which were subsequently used for SERS probe biomarker detection.^106,107^ Gold nanoparticles with a Raman reporter and a silica shell containing reactive thiols were functionalized with fluorophores with different excitation wavelength in addition to monoclonal antibodies (mAbs) *via* NHS–PEG_*n*_–maleimide, and these platforms were applied in the molecular imaging of fresh tissue surfaces.^108–110^ In summary, maleimide–thiol coupling has provided a versatile and effective method for covalently attaching molecules to the surface of core–shell nanoparticles.

### 2.5 Other methods

**Fig. 15:**
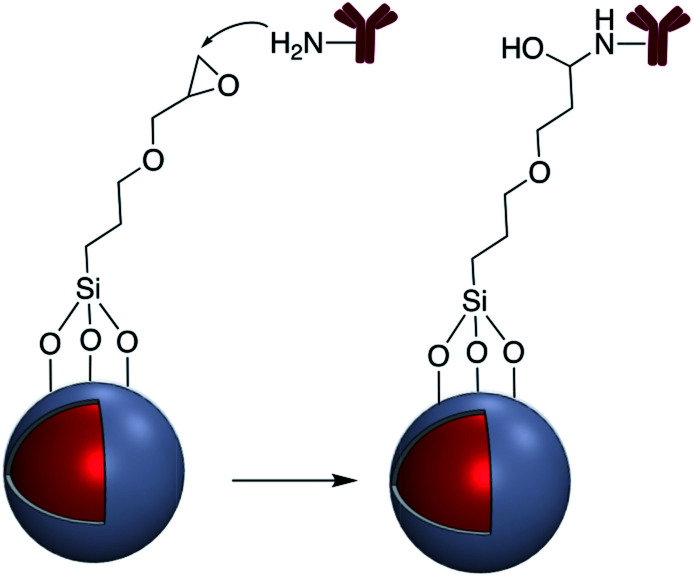
Antibody reaction with epoxy-activated M@SiO_2_ (M = Ag, Au).

In addition to the strategies described in the preceding sections, there have been some other methods used for the modification of core–shell nanoparticle surfaces. For example, (3-glycidoxypropyl)triethoxysilane, which contains a reactive epoxy group was used for thiol, amine, or hydroxy-conjugation onto the surface of a SiO_2_ shell. According to Krishnan *et al.* anti-*E. coli* McAb was immobilized onto the surface of Ag@SiO_2_*via* an epoxy-amino reaction and this bioconjugate was used for the sensitive detection of *E. coli* bacteria cells. The surface of the nanoparticle was functionalized with (3-glycidoxypropyl)triethoxysilane prior to linking to the antibody as shown in Fig. 15.^111^

**Fig. 16:**
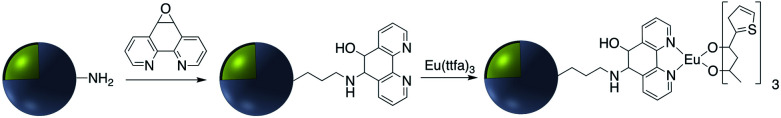
Preparation of luminescent Ag@SiO_2_ NPs coated with europium chelates.

Zhang and co-workers investigated the covalent binding of a europium chelate to the surface of Ag@SiO_2_ core–shell NPs using epoxide chemistry. In this report the surface of amine-functionalized nanoparticles was modified with the phenanthroline derivative epoxyphen. Eu(ttfa)_3_ was then used to insert europium(iii) ions into epoxyphen on the surface of Ag@SiO_2_ and the resulting luminescent core–shell NPs were used in optical multiplexing applications (Fig. 16).^112,113^

**Fig. 17:**
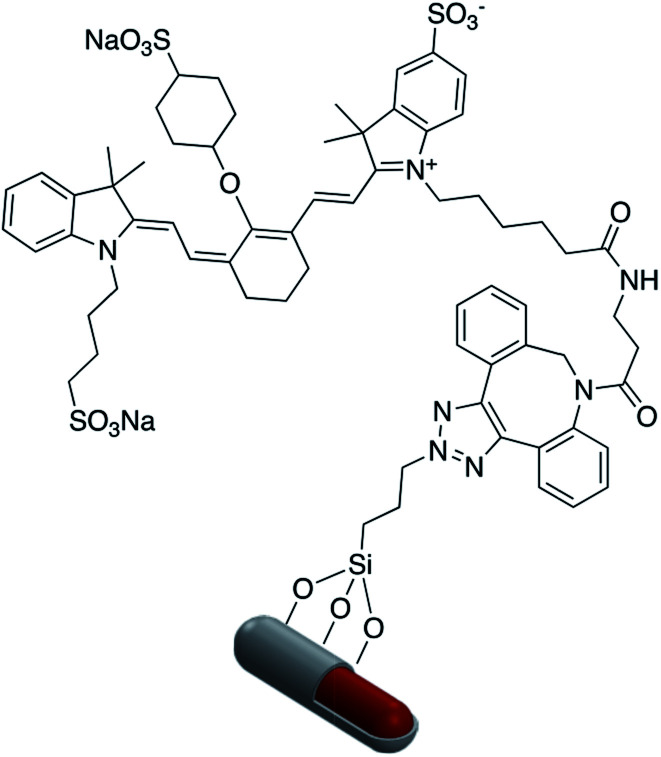
AuNR@SiO_2_ modified with IRDye 800CW DBCO.

“Click” chemistry has also been used for surface attachment of molecules. For example, Abadeer *et al.* covalently functionalized the core–shell Au-nanorod@SiO_2_ with an organic azide for further conjugation to IRDye 800CW DBCO *via* a copper-free click reaction (Fig. 17).^114^ “Click” chemistry is a well established method in organic synthesis used for the one-pot assembly of substrates into a desired target molecule. The classic “click” chemistry reaction involves a copper(i) catalyzed azide–alkyne cycloaddition reaction to produce a 5-membered triazole ring.^115^ It is not surprising that this method has been utilized for the grafting of molecules to the surface of core–shell nanoparticles. Asselin and co-workers have reported the application of “click” chemistry for the attachment of fluorescent Ag@SiO_2_–FITC nanoparticles (FITC = fluorescein isothiocyanate) to a silica surface.^14^ This assembly provided a method for determining extracellular pH using quantitative fluorescence.

**Fig. 18:**
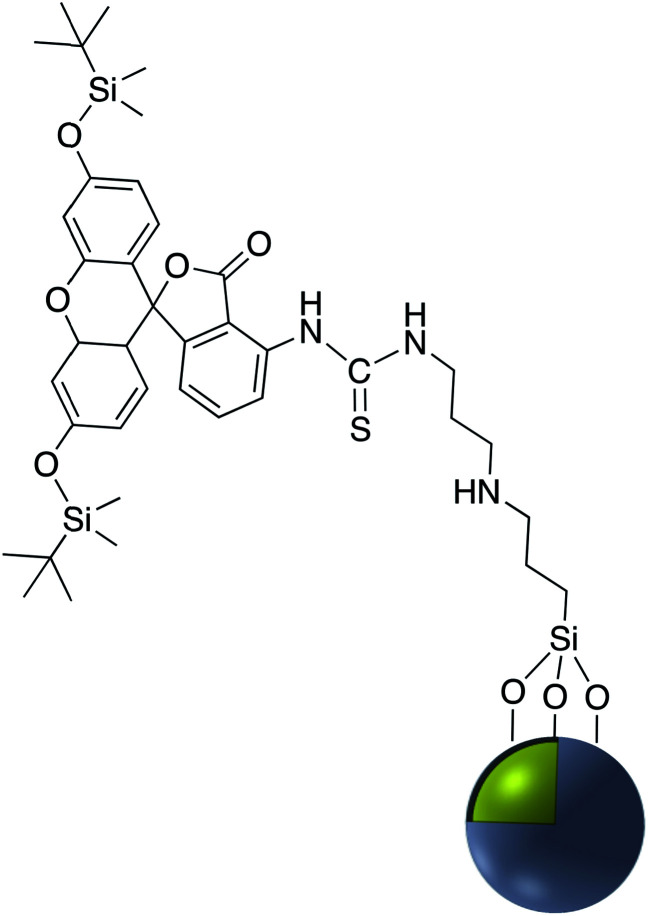
A core–shell metal-enhanced fluorescence biosensor.

Fluorophore labeled Ag@SiO_2_ NPs were synthesized *via* a simple reaction of the cyanate group in rhodamine B isothiocyanate (RITC) with amine-functionalized core–shell nanoparticles and the resulting platform used to determine the effect of shell thickness on fluorescence enhancement of the silver core. The hydroxyl groups of Ag@SiO_2_ were salinized with *N*-ethylenediamine (TMPED) to provide terminal primary amines on the surface for subsequent reaction with RITC. The last step was protecting the free phenolic OH with *tert*-butyldimethylsilyl chloride (TBDMS). The resulting probe was used as a metal enhanced fluorescence (MEF) biosensor (Fig. 18).^116,117^

In addition to the use of the –OH group for attachment of molecules, SH-functionalized Ag@TiO_2_ nanoparticles have also been used for surface attachment. Quantum dot (QD) – decorated Ag@SiO_2_ nanoparticles into which a Raman reporter *p*-aminothiophenol (PATP) was embedded, were synthesized and used for simultaneous SERS and SEF-based immunoassay.^118^ This technique was also used to prepare CD47 antibody-SERS nanoparticles for the identification of breast cancer.^119^ Immobilization of various vitamins including nicotinic acid, pantothenic acid and biotin on the surface of amine-modified Ag@SiO_2_ was accomplished using *N*-ethoxycarbonyl-2-ethoxy-1,2-dihydroquinoline (EEDQ) as a coupling agent.^120^ Europium complexes were grafted onto the surface of Au@SiO_2_*via* the condensation of siloxane groups of *N*-(4-benzoicacidyl), *N*-(propyltriethoxysilyl) urea (RABI) followed by further reaction with europium(iii).^121,122^ Xu *et al.* developed core–shell fluorescent nanoparticles Ag@SiO_2_@SiO_2_–RuBpy that were used for the fluorescence detection of prostate specific antigen (PSA) which is a tumor marker for the diagnosis of prostate cancer.^35^ In this report RuBpy were encapsulated in the outer silica layer of the nanoparticles. The fluorescent nanoparticles had the highest photoluminescence enhancement when the distance between the surface of silver core and the center of the RuBpy doped silica shell was about 10 nm. Ribeiro *et al.* modified Au@SiO_2_ NPs with a PDI (perylenediimide) dye bearing an alkoxysilane moiety that reacted with the silanol groups of the silicon oxide shell. Enhancement of the dye emission was subsequently analyzed.^123^ Phenylazathiacrown molecules possessing a silane group were anchored to Au@SiO_2_ NPs, and this platform was used for SERS detection of mercury ions.^124^ The same year, Yan *et al.* investigated the fluorescence-enhancing effect of Ag@SiO_2_ NPs with fluorescein molecule (FITC) doped silica *via* a high throughput single-particle analysis.^125^ Samarium and dysprosium benzoate complexes were grafted to the surface of Ag@SiO_2_ NPs and their luminescence properties studied^126^ and europium complexes grafted onto Au@SiO_2_ NPs were used for singlet oxygen detection.^127^ Using atom transfer radical polymerization, it was demonstrated that grafting 3-(triethoxysilyl)propyl 2-bromo-2-methylpropanoate (BMPS) onto Ag@SiO_2_ gave a platform onto which polymethylmethacrylate (pMMA) could be attached.^128^ The authors demonstrated that Au@SiO_2_@pMMA particles were able to produce a close-packed monolayer at the air–water interface and that this monolayer lost all plasmonic properties when compared to the free nanoparticles in solution.

**Fig. 19:**
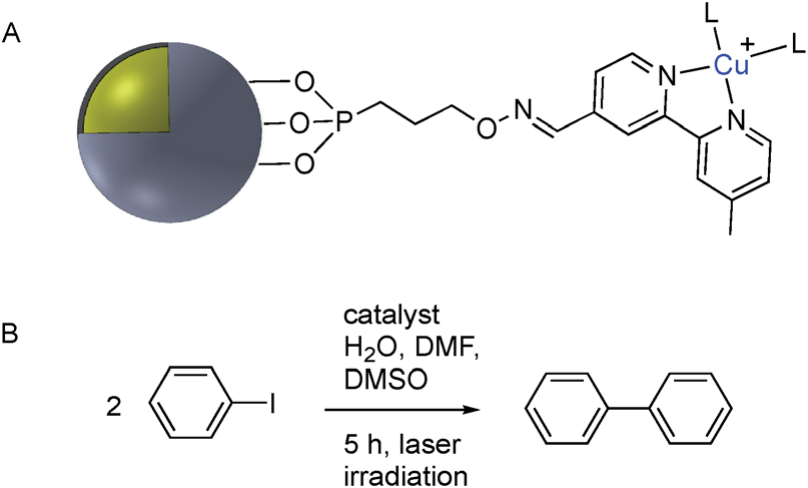
(A) Phosphonate functionalized core–shell Ag@TiO_2_ nanoparticle with pendant bipyridine–Cu(i) catalyst. (B) Cu(i) catalyzed Ullmann coupling reaction.

Although the use of silica as a coating for noble metal nanoparticles is by far the most wide-spread, a number of reports have described the covalent grafting of functionalized phosphonic acids to core–shell Ag@TiO_2_ and Ag@ZrO_2_ and use in a number of applications. Queffelec and Knight described a bipyridine–phosphonic acid and subsequent covalent attachment to 40 nm M@TiO_2_. Copper(i) was then complexed to the bipyridine coated nanoparticles and the resulting plasmonic nanomaterial used for the catalytic Ullmann reaction (Fig. 19).^129^ Irradiation of the Ag plasmon band with blue laser light at 488 nm resulted in homocoupling of iodobenzene to give biphenyl with a yield of 55% *vs.* <1% for the dark reaction. The same nanocomposite was also used as a sensor platform for detection of copper(ii) ions using SHINERS (Shell-Isolated Nanoparticle-Enhanced Raman Spectroscopy) at picomolar concentrations of Cu(ii) in water.^130^

## 3. Surface modification using electrostatic interaction

Electrostatic adsorption results from the interaction of either negatively or positively charged nanoparticles and a corresponding cation or anion. The major advantage of an electrostatic surface modification is the rate at which the surface bonding occurs relative to the analogous covalent modification. The main disadvantage of non-covalent binding is that the interaction is relatively weak and molecules adsorbed to the charged surface may be readily replaced by competing ions and molecules.

**Fig. 20:**
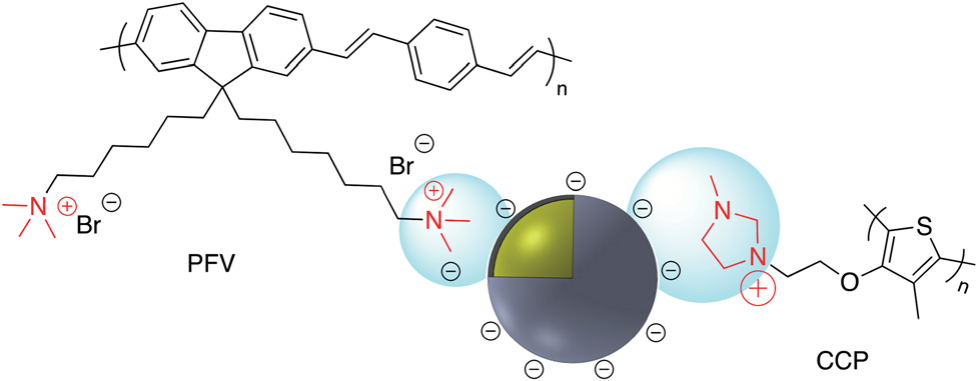
Positively charged conjugated polymers PFV and CCP on the surface of negatively charged Ag@SiO_2_.

Numerous examples of electrostatic surface modification have appeared in the recent literature. For example, positively charged conjugated polymers (CPs), bis(6-(bromohexyl)fluorene-2,7-ylenevinylene-*co-alt*-1,4-phenylene) (PFV) and poly(1*H*-imidazolium,1-methyl-3-[2-[(4-methyl-3-thienyl)oxy]ethyl]-chloride) (CCP) were assembled onto the surface of negatively charged Ag@SiO_2_ to give novel biosensors (Fig. 20).^131,132^

**Fig. 21:**
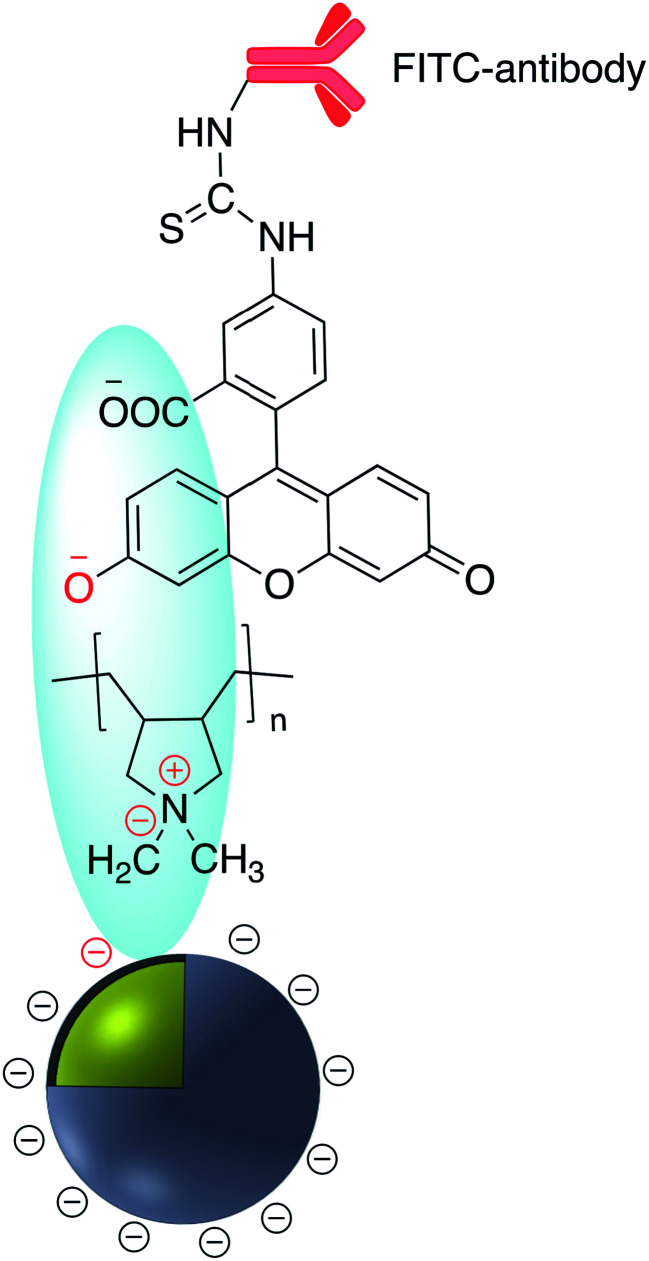
FITC-antibody modified Ag@TiO_2_.

**Fig. 22:**
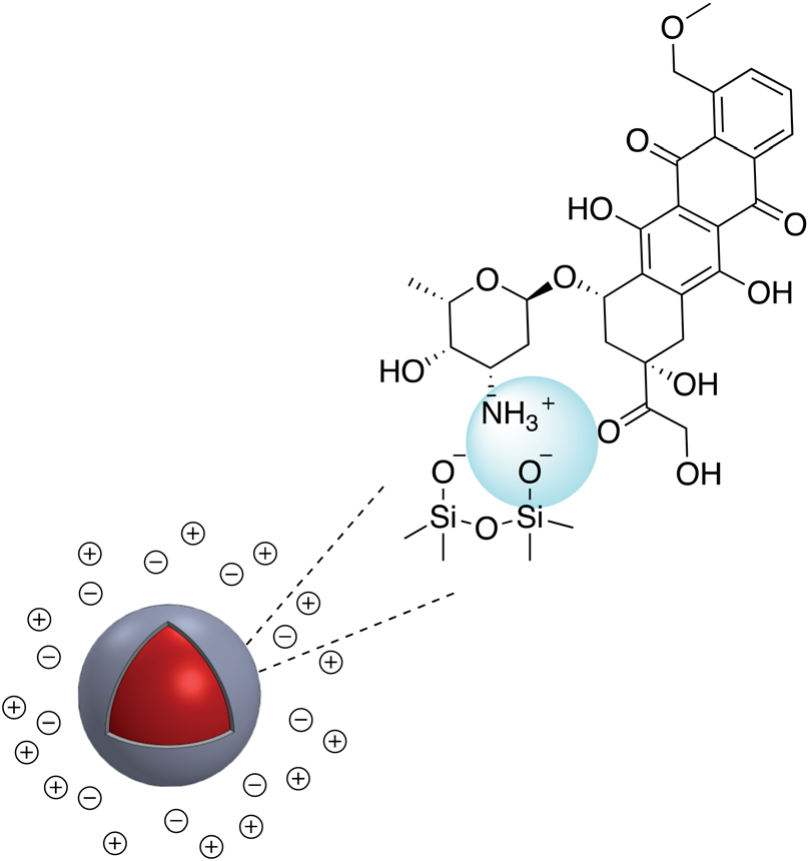
Au@SiO_2_ modified with doxorubicin.

According to Yang *et al.* poly(allylamine hydrochloride) PAH was used to electrostatically cover the surface of mesoporous silica coated AgNP which was previously impregnated with a fluorophore.^133^ Polydimethyldiallylammonium chloride (PDDA) was adsorbed onto the surface of negatively charged Ag@SiO_2_. Ag@SiO_2_@PDDA was further modified by the adsorption of IgG–FITC and subsequently used as a biomarker (Fig. 21).^134^ A similar method was used to immobilize the positively charged anti-cancer therapeutic doxorubicin hydrochloride (DOX), onto the surface of negatively charged Au@SiO_2_ (Fig. 22).^135,136^ The irreversible nature of the electrostatic adsorption of DOX makes it suitable for the facile release of the therapeutic in drug delivery applications.

DOX was also adsorbed on the surface of hollow/rattle mesoporous Au@SiO_2_ and showed high capacity for DOX loading.^137,138^ According to Sudeep *et al.*, Ag@TiO_2_ core–shell nanoparticle modified with the anionic tricarbocyanine dye (IR-125, Indocyanine Green) showed increased photoresponse and was applied to produce dye-sensitized solar cells.^139^ IR-125 was also adsorbed onto the surface of Au-nanostar@SiO_2_ and the conjugate exhibited significant fluorescence enhancement.^140^ Bai *et al.* coated Ag@SiO_2_ with 1-hydroxypyrene-3,6,8-trisulfonic acid (HPTS) *via* electrostatic absorption, and the pH sensitivity of the resulting probe has a practical application as a pH probe in biological systems.^141^ Levofloxacin hydrochloride (LEVO), a fluoroquinolone with a broad range of activity against both Gram-negative and Gram-positive bacteria was loaded onto the surface of mesoporous silica surface coated AgNP. LEVO has a zwitterionic molecular structure and can be loaded inside mesoporous silica through electrostatic interaction.^142^ The increase of Raman signals of pyrene due to the enhanced electric fields on the surface of the silver nanoparticles has been investigated by controlling the thickness of the silica shell. Shanthil *et al.* modified the surface of Ag@SiO_2_ with pyrene methylammonium (Py-A) by means of the strong electrostatic interaction between the negative surface charges of the core–shell nanoparticle and the cationic ammonium group of the pyrene derivative.^143^

**Fig. 23:**
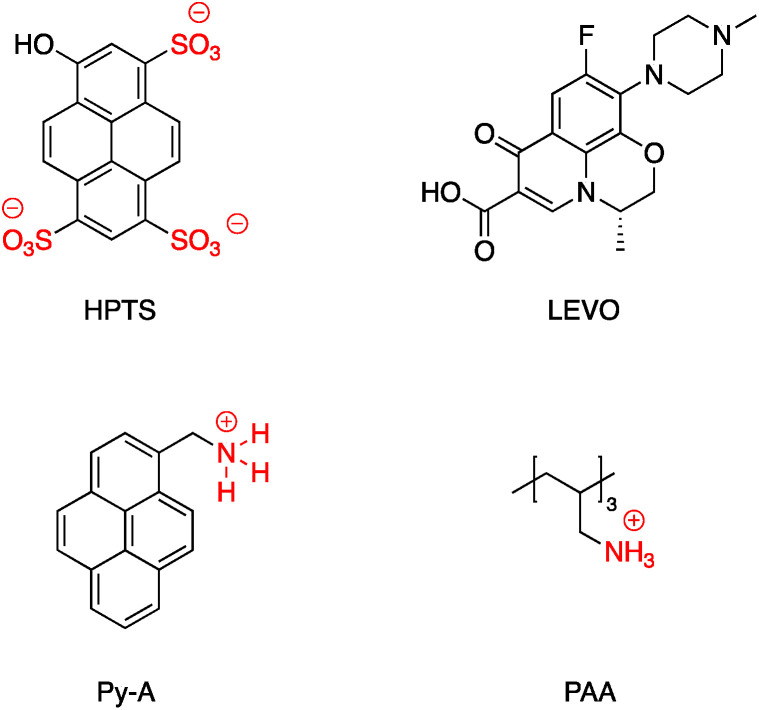
Examples of cations, anions and zwitterions used for functionalizing core–shell nanoparticles.

A SERS platform based on carboxyl-functionalized core–shell structure of Ag@SiO_2_ nanoparticles (NPs) was developed by Liu *et al.*^144^ in which negatively charged carboxylate functional groups were surface immobilized on the NPs, and it was shown that SERS sensitivity and stability were significantly improved towards positively charged biological and non-biological molecules. The sensitivity for the detection of molecules with positive –NH_3_^+^ groups such as basic orange and crystal violet was improved by two orders of magnitude compared to silver NPs, while detecting the Brilliant blue dye bearing the negatively charged SO_3_^−^ group was effectively suppressed by approximately about three orders of magnitude. The positively charged –NH_3_^+^ groups in APTES were also used for the assembly of APTES decorated SERS-active gold nanostars on an indium tin oxide (ITO surface)^145^ and amine groups on APTMS (3-aminopropyltrimethoxysilane)-functionalized Ag@TiO_2_ were used to prepare hybrid magnetic Ag@TiO_2_–Fe_3_O_4_ nanoparticles.^146^ Similarly, the cationic amine groups in poly(allylamine hydrochloride) (PAA) were used to attach Au@SiO_2_ NPs to graphene oxide^147^ and CdS QDs were attached electrostatically to Au@SiO_2_–NH_2_.^148^ Select examples of ions which have been electrostatically bound to core–shell nanoparticles are shown in Fig. 23. Fluorescence sensing platforms were prepared by electrostatic adsorption of Rhodamine 800 (Rh800) and Eu-TDPA [tris(dibenzoylmethane) mono(5-aminophenanthroline)-europium] onto the surface of Ag@SiO_2_ ^149,150^ and similarly, outer-shell periodic mesoporous organosilicas (PMOs) containing bis(rhodamine Schiff-base derivative) siloxane groups were grafted onto Ag-nanocube@SiO_2_ and used as a fluorescent sensor for Cu^2+^ ions. Hemoglobin (Hb)–Au-nanorod@SiO_2_ was prepared by a simple electrostatic interaction between a silica shell and Hb to further expand applications in hemoglobin electroanalytical studies.^151^ In another study, electrostatic interaction was used to attach antibody molecules onto silica-coated gold nanorods. The positively charged antibodies, including anti-HER2 (anti-C-erbB-2-HER2/NEU) and anti-CEA8 (anti-CEA8 cam8/CD67), were adsorbed onto the surface of the negatively charged core–shell nanoparticles for further application for early cancer diagnosis.^152^ Kobayashi *et al.^153^* modified the surface of positively charged Au@SiO_2_@NH_2_ by simply adding cellulose, and the final product was used in X-ray imaging and Kong and co-workers described the hydrogen bonding of europium and terbium coordination polymers based on *o*- and *p*-phthalic acid chelate on the surface of Ag@SiO_2_ core–shell nanoparticles. The resulting nanomaterial showed enhanced luminescence emission and was applied in biosensing and optical multiplexing.^154^ An *et al.* reported the modification of the surface of positively charged gold nanorods coated with SiO_2_ using Indocyanine Green (ICG, negatively charged) and DOX (positively charged). Nanocomposites which were further covalently bonded to the folic acid modified GNRs@mSiO_2_–ICG–DOX@FA were used in chemo-/photothermal/photodynamic therapy under near-IR irradiation.^155^ Although the majority of examples for electrostatic binding reported in the literature highlight the use of silica, non-covalent modification of core–shell titania coated nanoparticles has also been described. For example, *in situ* electrochemical SERS properties of the AgNP core in Ag@TiO_2_ were used to probe the interaction between N719 dye and an oxide shell. In this example, the N719 dye was chemisorbed to the TiO_2_ surface by simply immersing the nanoparticles in an aqueous dye, followed by rinsing off excess dye. Electrochemical SERS studies showed that core–shell nanoparticles have a bell-shaped potential dependence of the intensity for the Raman bands of the N719 ruthenium-based dye, which the authors explain is likely due to photon-induced molecule-to-TiO_2_ charge-transfer resonance.^156^ A related dimeric core–shell Ag@TiO_2_ nanoparticle system was also used to study the off-resonance Raman characteristics of the Ag@TiO_2_ interface.^157^ Finally, a single example of ligand stabilized Ag@ZrO_2_ and Au@ZrO_2_ core–shell nanoparticles has been described. In this report stearic acid was used to coat the zirconia surface of the nanoparticles which were formed *via* reduction of the corresponding metal salts in the presence of stearic acid. The resulting hydrophobic nanoparticles were found to be fully dispersible in a variety of polar and non-polar solvents.

## 4. Summary and perspectives

Metal/metal oxide core–shell nanoparticles with plasmonic noble metal cores and silica and titania shells have been surface modified and stabilized with a variety of biomolecules, simple organic molecules and metal complexes through both covalent binding using well known organic coupling chemistry, and non-covalent electrostatic interaction. Covalent attachment has been achieved through the direct reaction of chemical modifiers with functionalized core–shell nanoparticles or by the use of common bifunctional cross-linkers. Methods commonly used include amide and imine bond formation, cyanuric chloride activation, and maleimide–thiol coupling. Non-covalent binding has been used for surface modification of nanoparticles *via* electrostatic interaction of molecular ions with the charged nanoparticle surface. Using these methods, therapeutics and polymeric materials have been surface-attached. Taking advantage of the plasmonic core, these multifunctional nanostructures have been applied in a variety of different fields including laser-assisted homogeneous catalysis including copper catalyzed carbon–carbon bond formation and phosphate ester hydrolysis, biophotonics and sensing. Despite the wealth of metal@metal oxide core shell nanoparticles reported in the literature in recent years, extensive surface modification has so far been limited to noble metal@SiO_2_ and TiO_2_ platforms. This is surprising considering that both transition metal nanoparticle cores and corresponding oxides or main group metal and lanthanide metal oxide shells have potential in a number of contemporary applications including theranostics,^158^ water-splitting catalysis,^159^ highly ordered metallodieletrics^3^ and gas sensing.^18^ The development of novel surface modifiers with interesting properties is also a field ripe for exploration. For example chiral modifiers for plasmon assisted asymmetric catalysis is as yet very poorly explored. There exists an entire panoply of homogeneous catalytic reactions for which rate enhancements and selectivities may be improved using SPR assisted catalysis *e.g.* oxidation, reduction, C–N and C–O bond formation, and polymerization reactions. Such reactions will require the development of new ligands, metalloligands, core–shell platforms and corresponding surface modification techniques to be successfully realized.

## Conflicts of interest

There are no conflicts of interest to declare.

## Supplementary Material
